# What Is a Language? Who Is Bilingual? Perceptions Underlying Self-Assessment in Studies of Bilingualism

**DOI:** 10.3389/fpsyg.2022.863991

**Published:** 2022-05-12

**Authors:** Danika Wagner, Ellen Bialystok, John G. Grundy

**Affiliations:** ^1^Department of Psychology, York University, Toronto, ON, Canada; ^2^Department of Psychology, Iowa State University, Ames, IA, United States

**Keywords:** language, dialect, written language, bilingual experience, degree of bilingualism

## Abstract

Research on the cognitive consequences of bilingualism typically proceeds by labeling participants as “monolingual” or “bilingual” and comparing performance on some measures across these groups. It is well-known that this approach has led to inconsistent results. However, the approach assumes that there are clear criteria to designate individuals as monolingual or bilingual, and more fundamentally, to determine whether a communication system counts as a unique language. Both of these assumptions may not be correct. The problem is particularly acute when participants are asked to classify themselves or simply report how many languages they speak. Participants' responses to these questions are shaped by their personal perceptions of the criteria for making these judgments. This study investigated the perceptions underlying judgments of bilingualism by asking 528 participants to judge the extent to which a description of a fictional linguistic system constitutes a unique language and the extent to which a description of a fictional individual's linguistic competence qualifies that person as bilingual. The results show a range of responses for both concepts, indicating substantial ambiguity for these terms. Moreover, participants were asked to self-classify as monolingual or bilingual, and these decisions were not related to more objective information regarding the degree of bilingual experience obtained from a detailed questionnaire. These results are consistent with the notion that bilingualism is not categorical and that specific language experiences are important in determining the criteria for being bilingual. The results impact interpretations of research investigating group differences on the cognitive effects of bilingualism.

## Introduction

Most of the research investigating the cognitive and brain consequences of bilingualism relies on assigning participants to language groups. Some studies comparing groups based on these categorical designations have reported that bilinguals performed more accurately or faster than monolinguals on various cognitive tasks and measures, especially those related to executive functioning, whereas other studies found no differences between groups (summary in Antoniou, 2019). Both conclusions have been supported by meta-analyses that either confirm the reliability of the group difference (Adesope et al., 2010; Grundy and Timmer, 2017; van den Noort et al., 2019; Grundy, 2020; Ware et al., 2020; Monnier et al., 2021) or fail to reject the null hypothesis (de Bruin et al., 2015; Lehtonen et al., 2018; Donnelly et al., 2019; Lowe et al., 2021). One factor contributing to these conflicting results is the definition of “bilingualism” and how participants are assigned to groups in various studies (for discussion see Bak, 2016; Bialystok, 2016). As discussed by Luk and Bialystok (2013), bilingualism is not a categorical variable. Some current research avoids this problem of the non-categorical nature of bilingualism by treating bilingualism on a continuum and investigating the impact of the degree of bilingual experience on outcome measures (Gullifer et al., 2018; Pot et al., 2018; DeLuca et al., 2019, 2020; Gullifer and Titone, 2020; Kałamała et al., 2021). In most cases, however, these studies using continuous measures for bilingualism do not include monolinguals and so cannot address the underlying question regarding potential language group differences in performance. Nonetheless, the positive relationship between the degree of bilingual experience and cognitive outcomes in the continuous studies consists of the role of bilingual experience in reshaping cognition.

Despite this new direction, the majority of research on bilingualism compares performance across two binary groups. The categorical approach to defining groups is especially problematic in studies in which the group assignments are made by self-assessment by the participants. How reliable are individuals' self-descriptions of their own bilingualism? There are no consistent or objective standards that determine the point at which someone transitions into a category called “bilingual.” More concerning, before one can determine whether their mastery of a language is adequate to be described as “bilingual,” there needs to be agreement on what counts as a “language.” Again, the criteria are less transparent than one might believe. Although the linguistic distinction between language and dialect is an ongoing topic of inquiry for researchers (Melinger, 2018, 2021), participants in research studies are unlikely to be aware of those discussions.

Anecdotal occurrences in our lab have revealed a substantial number of participants who self-reported to be members of one group but on further inquiry using a detailed questionnaire (Language and Social Background Questionnaire; Anderson et al., 2018) were found to belong to the other group. For example, potential participants who had signed up to participate in a study as “monolingual” reported during the language background interview that they knew a second language, often to a high degree of proficiency. When asked why they considered themselves to be monolingual, the most common responses were, “Well, that language does not really count,” or “I only use it at home.” Similarly, other participants declared themselves to be “bilingual” but were found to have very less proficiency in the other language or were reporting a language they had briefly studied in school. Relatedly, Kirk et al. (2021) investigated language switching among speakers of Standard Scottish English (SSE) and two regional Scottish dialects of English (Orcadian and Dundonian) and found that most participants would be considered monolingual if language experience was measured using a language use questionnaire. This is because most participants, particularly those who spoke SSE and Orcadian, viewed the regional dialect as a way of speaking, rather than as a language, as it is closely related to English. Therefore, reliable judgments about bilingualism require a clear notion of what counts as a language.

In the absence of a consistent set of criteria for what counts as a language and how much experience is necessary for bilingualism, the group structures in most of this study lack validity, and conclusions that follow from those studies may be incorrect. For example, in a large-scale study by Nichols et al. (2020), the researchers concluded that bilingualism afforded no general cognitive advantage. Participants in that study were classified into two language groups based on their answers to a single question about how many languages they spoke without quantifying or qualifying their ability or use of that language, a procedure that is inadequate for assessing language experience (Luk et al., 2021). Similarly, studies that base group assignment on self-assessment of proficiency (e.g., Paap and Greenberg, 2013; Paap and Sawi, 2014) risk creating groups that fail to reflect relevant differences in bilingual experience. Without clear distinctions about what level of proficiency is needed to be perceived as bilingual and which languages should be considered in the calculation, the criteria for defining the category remain an open question.

Decisions about one's own language proficiency and bilingualism interact with sociolinguistic factors. Tomoschuk et al. (2019) asked Spanish-English and Chinese-English bilingual participants to classify themselves as either balanced bilinguals in that they were equally proficient in both languages or dominant bilinguals in that one language was stronger. Objective tests of proficiency were given to participants in both groups. However, participants whose objective language scores indicated balanced proficiency nonetheless claimed to be dominant in one language. Moreover, the results differed somewhat between the two bilingual groups. Chinese bilinguals used more extreme ratings to describe their own proficiency than the Spanish bilinguals, even when the objective scores were comparable. These results indicate that the bilinguals in the study were either not sufficiently aware of their own proficiency or lacked a definition of the criteria for the balanced vs. dominant categories and that the judgments interacted with the language group, underlining the unreliability of self-assessment. Therefore, addressing the defining conditions for a language and standards for bilingualism precedes resolving the contradictory evidence for the effect of bilingualism on cognitive outcomes. This study investigates the criteria people use when determining what counts as a language or deciding whether an individual is bilingual. Our purpose is not to identify formal linguistic criteria for these concepts, but rather to uncover the assumptions that influence participants when making self-assessments of bilingualism.

Broadly speaking, a language is a structured system of communicating sounds or signs that convey meaning. However, languages differ from each other in important ways, and it is not clear which of these differences are essential to deciding that the system is an independent “language.” For example, nearly half of the world's languages have a writing system (Sandler, 2018), but some languages, such as Creole and many Indigenous languages, lack this feature. Is the presence of a writing system necessary for a spoken system to be considered a language?

Linguistic relatedness is also relevant to determining unique language status. For example, the group of Romance languages includes Spanish, French, Italian, Portuguese, and Romanian, all of which are derived from Latin and recognized as distinct but related languages. But what is the limit of similarity for a system to be a distinct language? Many languages include dialectic variations, some of which are spoken only in geographically specific areas. For example, Flemish is a dialect of Dutch spoken only in the Flanders region of Belgium, and Swiss German is a dialect of German spoken only in a region of Switzerland. Are these languages distinct? To what extent do relatedness to another language and geographic specificity determine whether a system is an independent language? Although some researchers have investigated the cognitive effects of bidialectalism (e.g., Lundquist and Vangsnes, 2018; Poarch et al., 2019; Vorwerg et al., 2019; Melinger, 2021), there is no objective standard for determining when a dialect becomes a language.

Similarly, the determination of bilingualism varies across individuals and linguistic contexts (Baum and Titone, 2014; Kroll et al., 2018; Fricke et al., 2019; Kremin and Byers-Heinlein, 2021). These features include proficiency (Rosselli et al., 2016; Tomoschuk et al., 2019), quantity and quality of use (Hofweber et al., 2016; Hartanto and Yang, 2019; Gullifer and Titone, 2020), age of acquisition (Luk et al., 2011; Birdsong, 2018; Gullifer et al., 2018; Hernandez et al., 2018; Bylund et al., 2021), simultaneous (i.e., two languages from birth or at a very young age) or sequential (i.e., learning a second language after significant exposure to a first language) language learning (Brito et al., 2016; Delcenserie and Genesee, 2017; Kousaie et al., 2017), and passive vs. active bilingualism (Hartanto and Yang, 2019). As individual bilinguals have developed different skills to different levels, the boundaries for determining whether an individual is bilingual are unclear.

Surrain and Luk (2019) discussed the lack of a clear definition of bilingualism in a review of the literature on the labels used by researchers to describe bilinguals and monolinguals. They examined 186 studies and reported that 31% of them referred to bilinguals without any qualifiers explaining their linguistic profiles. Although most studies reported language proficiency (77%) or usage (79%), other linguistic experiences, such as the age of language acquisition, language learning status, simultaneous or sequential bilingualism, and sociolinguistic context, were not reported. Surrain and Luk (2019) concluded there is no clear definition of what constitutes bilingual experiences, or which features of those experiences are most important. Therefore, it should not be surprising that participants are potentially less reliable than researchers in making these judgments.

If bilingual experience is considered more broadly, then it extends to individuals who are usually considered to be monolingual (Leivada et al., 2020). Bice and Kroll (2019) reported that passive exposure to a multilingual environment influenced language processing in monolinguals, underlying the importance of group classifications in interpreting results. For example, Prior and MacWhinney (2010) compared monolinguals and bilinguals and found smaller switching costs for bilinguals, but Hernández et al. (2013) replicated the study and found no group difference. However, in the Prior and MacWhinney study, bilinguals had learned both languages before the age of 6 years and used both continuously ever since, but in the Hernandez study, the “monolinguals” self-reported proficiency in a foreign language as 2 on a 4-point scale, in which 2 indicated sufficient proficiency to deal with basic activities. In other words, the studies were not the same.

Standard language ideologies are commonly shared beliefs among individuals who speak a language about how that language “should” be spoken (Forsberg et al., 2020), and these notions can also influence judgments of languages and bilingualism. Forsberg et al. (2020) examined the association between standard language ideologies and self-ratings of language proficiency among bilinguals who spoke Swedish and one other non-dominant or minority language. Participants contextualized their Swedish proficiency within a standard language ideology framework and judged their abilities in terms of their perception of what an outside referee would consider proper speech (Forsberg et al., 2020). Accordingly, participants rated their Swedish proficiency more harshly than their heritage language. Therefore, these beliefs may influence an individual's perception of nonstandard languages and whether speakers of those languages are bilingual (Forsberg et al., 2020).

The contexts in which languages are used may also influence judgments of bilingualism. According to the adaptive control hypothesis model (Green and Abutalebi, 2013), there are three primary interactional contexts, namely, single language, dual language, and dense code-switching. In single language contexts, each language is used in a unique context; in dual language contexts, both languages are used in the same context but with different speakers; and in dense code-switching contexts, the languages are completely intermixed. The perceptions of bilingualism that arise from different home language and social use experiences have not been explored.

Other variations in bilingual experiences that could lead to different perceptions of bilingualism include education level and type, proficiency, and competence with a writing system in one or both languages. Formal second language education is one path to becoming proficient in a second language, but how much education is needed before an individual is perceived as bilingual remains unclear. Moreover, it is unclear if the time since second language education influences perceptions of bilingualism. For example, if someone attended a language immersion program in primary school, are they considered bilingual in adulthood or older age?

Finally, as with determining whether a system counts as a language, it is unclear if a bilingual must be proficient in the writing system of both languages to be considered bilingual. Individuals who speak two languages that have written forms (e.g., English and Spanish) may be perceived as more bilingual than individuals who speak two languages in which only one can be written (e.g., French and Creole). This feature may explain why some participants believe that their language “does not count” in their self-assessment of bilingualism.

This study investigated the criteria by which participants determine whether a system qualifies as a unique language and the standards for deciding whether an individual is bilingual. These questions are implicit in all studies that ask participants to self-determine whether they are monolingual or bilingual. Participants were asked to judge the extent to which a fictional description of a linguistic system constituted a unique language, and the extent to which a fictional description of an individual's linguistic experiences qualified that person as bilingual. Therefore, there were two questions as follows: What is a language? Who is bilingual? For “What is a language,” the scenarios manipulated the presence of a writing system, relatedness to another language, and geographic isolation of the spoken language. For “Who is bilingual,” the descriptions manipulated patterns of language use, proficiency levels, education in a second language, and the presence of a writing system in both languages. Many studies have examined the effect of these variables on outcomes. For example, studies have compared individuals who speak two standard languages with those who speak a standard language and a dialect (Antoniou et al., 2020), or compared bilinguals who vary in education and age (Bialystok et al., 2005). Our question is not to investigate the impact of these variables on performance but rather to identify the extent to which these variables bias participants' judgments about what counts as a language and who can be considered bilingual. Since so much research relies on those judgments, it is important to understand their basis.

## Methods

Participants were students at York University who completed the study for course credit or community volunteers who were entered into a gift card draw. The study was administered online to 856 participants, but as explained below, the final sample consisted of 528 participants. Participants completed the Language and Social Background Questionnaire (LSBQ; Anderson et al., 2018) and responded to 26 fictional scenarios.

The LSBQ is a detailed questionnaire designed to assess bilingualism in diverse populations. It contains three sections of questions that yield participants' demographic information, self-assessments of language proficiency, and self-reported language use patterns. The results are submitted to a calculator to produce three factor scores, namely, non-English home use and proficiency, non-English social use, and English proficiency, which are then weighted to yield a continuous measure of the overall degree of bilingualism. The composite scores were scaled using the *scale* function for R (R Core Team., 2021) to produce a value between 0 and 8, with higher scores indicating more bilingual experience. This score has been shown to relate to the degree of cognitive outcome in both children (Bialystok and Shorbagi, 2021) and adults (DeLuca et al., 2019).

In addition to completing the LSBQ, participants were asked to self-identify as monolingual or bilingual. This classification produced two groups consisting of 157 monolinguals and 371 bilinguals who spoke English plus one of 59 other languages. Considering all participants, 91.5% were residents of Canada, 4% were residents of the United States, and 4.5% were residents of various other countries. Of those Canadian resident participants, most resided in Toronto, a diverse metropolitan city. Of those participants who were not Canadian residents, 82% self-classified as bilingual, most of whom were Spanish-English bilinguals.

The study was conducted online using Qualtrics (2019) (https://www.qualtrics.com). Potential participants gave informed consent before completing the LSBQ and rating the 26 fictional scenarios. All languages were given fictional names, such as “Sloblinch,” to remove potential biases against actual languages. To be included in the final analyses, participants had to pass a manipulation check that was presented at a random point in the fixed sequence of scenarios in which they were simply asked to press “2” on this trial. In total, 328 individuals failed the manipulation check and were excluded from further analyses, leaving 528 participants in the final sample (409 females, 103 males, 9 not specified) ranging in age from 18 to 83 years (*M* = 24.25, *SD* = 9.99). All procedures were approved by York University's Office of Research Ethics.

### What Is a Language?

To address the question “what is a language,” participants were asked to rate 6 fictional language scenarios on a scale from “Not a Language” (0) to “Language” (10). The 6 scenarios reflected the following three binary dimensions of language: presence or absence of a writing system; relatedness to another known language; and geographic specificity, that is, whether the language was confined to a particular region since purely regional languages might be considered dialects.

Each scenario differed from the others on only one dimension but provided information on at least two of the dimensions. For example, a scenario might describe a system that is written and related to another language that could be compared to a language that was not written and related to another language, isolating the impact of written language on judgments. To illustrate, a scenario featuring a “related” fictional language with a writing system says, “You are shopping in the grocery store and hear someone speaking Dostinese. Dostinese is similar to English but is written using a different writing system. Individuals who speak Dostinese can also understand and speak English because of the similarities. Is Dostinese a language?” This scenario could be compared to one that changes the value only for the writing system. Contrasts between scenarios that differed in a single feature allowed for the assessment of the role of that element. To summarize, the factors manipulated in these scenarios are the presence of a writing system, relatedness to another language, and geographic specificity. The scenarios are presented in Appendix A.

### Who Is Bilingual?

To address the question “who is bilingual,” participants rated 20 fictional language use and proficiency scenarios on a scale from “Monolingual” (0) to “Bilingual” (10). Each scenario highlighted a dimension, including level and type of education, time since second language education, continued use of both languages, proficiency in both languages, presence of a writing system in one or both languages, and various social use scenarios.

The role of education and experience in judgments of bilingualism were manipulated by describing young adults or middle-aged adults who had undergone one of the following three language education programs: core-language education, immersion education from elementary through secondary school, or extended immersion education into post-secondary education. Two analyses were conducted, one examining the time since second language education (age) and the second examining the type of education. For the young adult level, the scenario described an individual who was between the ages of 20 and 25 who had recently participated in one of the education programs. For the middle-aged adult, the individual was described as being between the ages of 39 and 45 years and had participated in one of the education programs in the past and had not used those languages in a long time. The name of the individual described in the scenario and the name of the fictional language were counterbalanced across scenarios. For example, the core education young adult scenario states: “Imagine an individual grew up speaking Jantsi in the home and the community but from the ages of 6 to 14 received daily, 1-h lessons in Gronk at school. This individual is now 21 years old. To what extent is that individual bilingual?” The middle-aged adult version of this scenario calls the fictional language that is taught “Brakien,” and the fictional individual is 48 years old. These comparisons allowed evaluation of the role of level and type of education and time education on the judgments of bilingualism.

As proficiency is obviously relevant to judgments of bilingualism, three levels of proficiency were compared as follows: low proficiency, in which the individual could only produce a few words in a second language; moderate proficiency, in which a person could speak in a limited capacity such that they defaulted to some words in their native language; and high proficiency, in which a person could speak a second language fluently.

Patterns of usage across various settings have been identified as a significant factor in bilingual experience. Therefore, the scenarios included three levels of community use patterns as follows: less usage in which remnants of a heritage language are spoken in the community; medium usage in which a heritage language is spoken in the community but not at home or school; and high usage that is similar to medium but includes extra-curricular instruction in that language.

Other scenarios manipulated usage patterns in the extended family and with close relatives. The first scenario described an individual who speaks one language at home but once a week the grandmother visits to teach them how to cook, an activity carried out in a second language. In the second scenario, the fictional individual spoke one language at home but spoke to their extended family members once a week on the phone in a second language. Finally, two scenarios described experiences with active or passive receptive language use. In the active scenario, the individual's parents speak to the individual in one language and the individual responds in another. In the passive scenario, the individual's parents speak one language to each other, exposing the individual to the language, but the family speaks a different language.

In another pair of scenarios, the fictional individual either continued to use their heritage language after immigration or not. In both scenarios, the individual immigrated to a new country where a new language was spoken later in life, after about 50 years of age. In the first scenario, the individuals discontinued using their heritage language instead of focusing on using the language of the new country they called home. In the second scenario, the individuals continued to use their heritage language while also learning the language of their new country. These scenarios allowed insight into the role of length of bilingual experience and continuation of bilingual language use in shaping perceptions of bilingualism.

The final factor was the impact of a writing system on judgments of bilingualism. This was examined by comparing two scenarios in which the second language had a written form or not.

To summarize, the factors manipulated in these scenarios were type and level of second-language education, time since second-language education, proficiency, community language use patterns, receptive language exposure, language use with extended family, language use after immigration later in life, and the presence of a writing system in both languages. A complete list of the scenarios is presented in Appendix B.

## Results

### Who Are the Participants?

**Figure 1:**
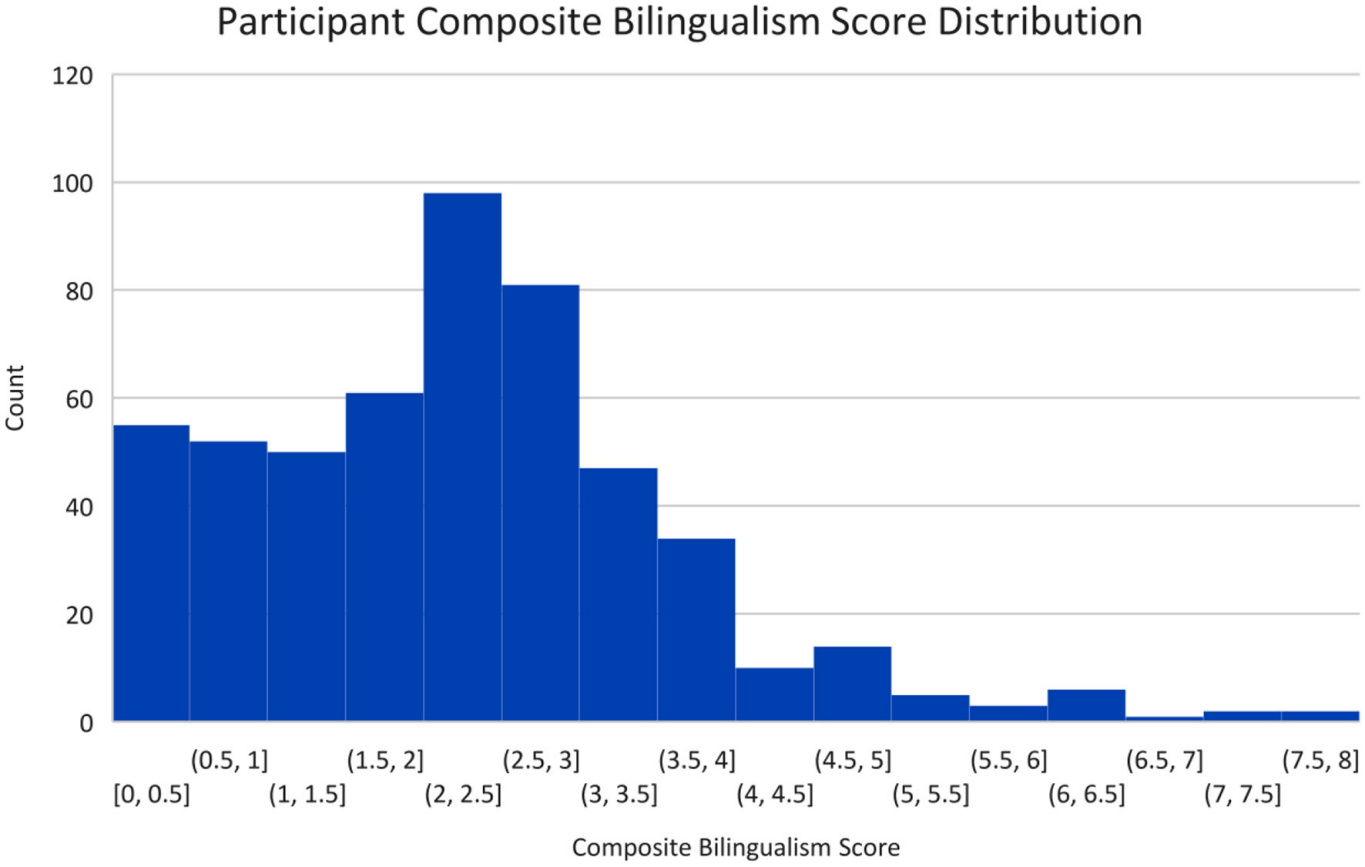
Composite bilingualism scores (ranging from 0 to 8) derived from the LSBQ.

As there were no language restrictions for participating in the study, the sample included individuals with a range of language experiences. The LSBQ composite scores were used to test the reliability of the self-classification of participants into two groups. The distribution of composite scores is shown in Figure 1. Scores ranged from 0 to 8 (*M* = 2.3, *SD* = 1.4), with the mean score for self-classified monolinguals as 1.02 (*SD* = 1.2) and for self-classified bilinguals as 2.8 (*SD* = 1.1). This difference was not statistically significant, *t*_(526)_ < 1, *ns*.

### What Is a Language?

Mean scores for each of the features embedded in the language scenarios are presented in Tables 1A,B. Because not every feature could appear in combination with all other features, standard parametric analyses were not possible, so targeted analyses were needed. Therefore, scores were examined in two 2 × 2 repeated measures ANOVAs. The first analysis evaluated scores for the four scenarios that manipulated the role of writing and relatedness, excluding geographic specificity. The second analysis evaluated scores for the four scenarios that evaluated writing and geographic specificity, excluding relatedness. Since the geographically specific language scenarios were designed to measure the perception of dialects, the languages in these scenarios were necessarily related.

**Table 1A:** Mean score out of 10 (standard deviations) for the extent to which the description indicates a unique language, comparing the presence of a writing system and relatedness to another language.

	**Written**	**Unwritten**	**Mean**
Related	8.3 (2.3)	7.1 (2.9)	7.7 (2.6)
Unrelated	8.6 (2.4)	7.7 (2.9)	8.2 (2.7)
Mean	8.5 (2.4)	7.4 (2.9)	

**Table 1B:** Mean score out of 10 (standard deviations) for the extent to which the description indicates a language comparing the presence of a writing system and geographic specificity.

	**Written**	**Unwritten**	**Mean**
Geographically broad	8.3 (2.3)	7.1 (2.9)	7.7 (2.6)
Geographically specific	7.9 (2.7)	7.1 (3.0)	7.5 (2.9)
Mean	8.1 (2.5)	7.1 (2.9)	

The two-way ANOVA for writing system and relatedness revealed a main effect of a writing system, *F*_(1, 527)_ = 115.77, *p* < 0.001, with written languages receiving higher scores than unwritten languages, the main effect of relatedness *F*_(1, 527)_ = 20.59, *p* < 0.001, with unrelated languages receiving higher scores than related languages, and an interaction between them, *F*_(1, 527)_ = 5.3, *p* = 0.02. Follow-up contrasts showed that for both related, *F*_(1, 527)_ = 100.2, *p* < 0.001, and unrelated languages, *F*_(1, 527)_ = 58.8, *p* < 0.001, language ratings were higher for written languages than unwritten languages. Similarly, the effect of relatedness was significant for both written, *F*_(1, 527)_ = 6.15, *p* = 0.01, and unwritten languages, *F*_(1, 527)_ = 24.3, *p* < 0.001. Therefore, the interaction effect is most likely caused by the larger difference between written and unwritten languages in the related scenario (0.59) than in the unrelated scenario (0.34), suggesting that relatedness matters more for unwritten languages than for written ones.

Similarly, a 2-way ANOVA was conducted for geographic specificity and the presence of a writing system. Since all of the languages were related in this case, the values for the geographically broad languages are based on the same scenarios as those reported for related languages in the previous analysis. There was a main effect of writing system, *F*_(1, 527)_ = 127.68, *p* < 0.001, an interaction between geographic specificity and writing system, *F*_(1, 527)_ = 7.04, *p* = 0.008, but no main effect of geographic specificity, *F* < 1, *ns*. Follow-up analyses revealed a significant difference between written and unwritten language scores for both the geographically broad, *F*_(1, 527)_ = 100.2, *p* < 0.001, and geographically specific conditions, *F*_(1, 527)_ = 71, *p* < 0.001. However, the contrast for geographic specificity was only significant for the written condition, *F*_(1, 527)_ = 7.79, *p* = 0.005, not for the unwritten condition, *F* < 1, *ns*.

Finally, correlations were calculated to determine if overall language scores were related to participants' composite bilingualism score, *r*_(526)_ = 0.08, *ns*, age, *r*_(526)_ = −0.02, *ns*, or education, *r*_(526)_ = 0.008, *ns*. None of the correlations were significant.

### Who Is Bilingual?

**Figure 2:**
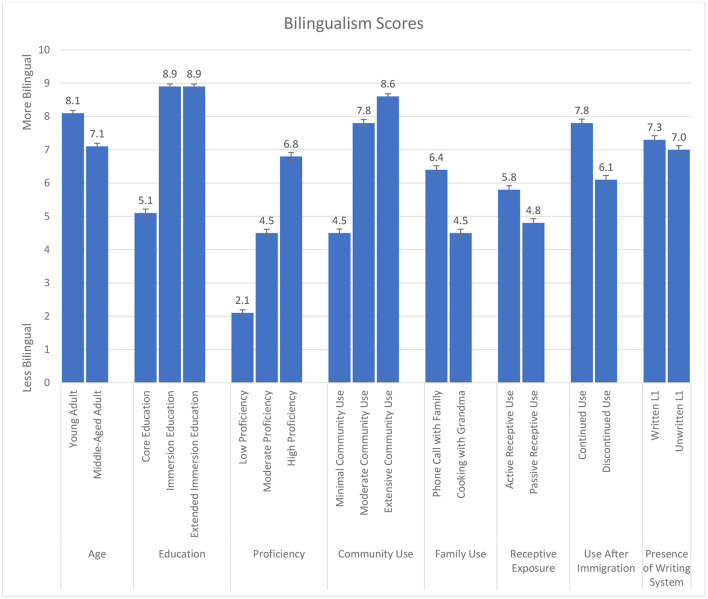
Bilingualism scores (out of 10) illustrating the impact of each manipulated variable on the designation that an individual is bilingual.

The mean bilingualism scores for each manipulated variable are presented in Figure 2. Again, as it was not possible to conduct multifactor ANOVAs with interaction terms, levels within each category were examined by one-way ANOVAs. First, a one-way ANOVA comparing two age groups (young adult and middle-aged) was conducted to examine the effects of time since second language education. The effect of age was significant, *F*_(1, 527)_ = 268.81, *p* < 0.001, with young adults classified as more bilingual than those who are now in middle age.

The three types of education programs (core education, immersion education, and extended immersion education) were significantly different, *F*_(2, 1054)_ = 919.36, *p* < 0.001. Fictional individuals who had taken immersion and extended immersion education, *F*_(1, 527)_ = 351.58, *p* < 0.001, were perceived as more bilingual than those who learned a second language through core education, *F*_(1, 527)_ = 273.29, *p* < 0.001. Not surprisingly, therefore, immersion experiences lead to higher perceived bilingual scores than core language education.

A one-way ANOVA for the three proficiency levels on judgments of bilingualism indicated a significant effect, *F*_(2, 1054)_ = 703.35, *p* < 0.001. All contrast intervals were significant: high proficiency was considered more bilingual than moderate proficiency, *F*_(1, 527)_ = 295.24, *p* < 0.001, and moderate proficiency was more bilingual than low proficiency, *F*_(1, 527)_ = 450.35, *p* < 0.001.

The differences in usage patterns indicated by three levels of community use showed a significant difference between them, *F*_(2, 1054)_ = 527.49, *p* < 0.001. Individuals who engaged in extensive community use were considered more bilingual than those who engaged in moderate use, *F*_(1, 527)_ = 105.45, *p* < 0.001, and moderate users were perceived to be more bilingual than minimal users in the community context, *F*_(1, 527)_ = 878.62, *p* < 0.001.

For usage within the home, there was a significant difference between the two extended family language use scenarios, *F*_(1, 527)_ = 228.57, *p* < 0.001, with phone conversations with extended family receiving a higher bilingualism score than cooking with grandma. There was also a significant difference between the two receptive language use scenarios in the home, *F*_(1, 527)_ = 70.22, *p* < 0.001, with the active scenario receiving a higher bilingualism score than the passive scenario. Therefore, the productive use of language by responding to the parents confers a higher bilingualism score than simply listening to them.

Continued use of a heritage language after immigrating to a new country produced higher scores for bilingualism than did discontinued use scenarios, *F*_(1, 527)_ = 168.84, *p* < 0.001. This difference was significant despite both scenarios featuring a fictitious individual with decades of language experience, suggesting the importance of ongoing usage for perceptions of bilingualism.

Finally, the role of written language on perceptions of bilingualism was evaluated by comparing bilingualism scores for individuals whose two languages were both written or those for whom only one was written. There was a significant difference in which speakers of two written languages were perceived as more bilingual than those for whom only one language had a writing system, *F*_(1, 527)_ = 7.11, *p* = 0.008.

Correlations for overall judgements of bilingualism and participants' bilingualism composite score were next examined, *r*_(526)_ = −0.07, *ns*, age, *r*_(526)_ = −0.00003, *ns*, and education, *r*_(526)_ = 0.004, *ns*. Again, none of the correlations were significant.

## Discussion

This study explored the influence of several key characteristics on determining how individuals decide whether a system counts as a unique language and the extent to which individuals with different experiences can be considered bilingual. The results revealed that these characteristics have a substantial impact on how individuals arrive at these decisions, making self-classifications relating to languages and bilingualism multidimensional and complex. Judgments were not influenced by participant characteristics including the degree of bilingualism, age, or education. These results have wide implications for research that compares groups of monolinguals and bilinguals across the lifespan and makes conclusions in terms of the group designation. The answers to the questions “What is a language?” and “Who is bilingual?” are summarized below.

### What Is a Language?

Participants considered systems that were unrelated to other languages rather than related, written rather than unwritten, and spoken widely rather than geographically specific to be more language-like rather than their counterparts. Consider each of these dimensions in turn. The results for relatedness were in line with expectations that similar systems might be considered dialects of the same language rather than unique languages. However, the distinction between dialects and distinct languages is not clear (Gregory and Carroll, 2018). For example, while Portuguese and Spanish are distinct languages, they have considerable similarity and some mutual intelligibility, whereas Flemish is not considered to be a distinct language from Dutch, despite having several different linguistic properties. Despite being written in different alphabets (Cyrillic vs. Roman), Serbian and Croatian used to be considered a single language, Serbo-Croatian, but after political upheaval in Yugoslavia, they are now simply considered to be different languages. Clearly, the boundaries of similarity that determine whether a system is a unique language are more continuous than categorical.

A few studies have examined the effect of language similarity on cognitive outcomes of bilingualism, but the results are mixed and the conclusions at this point are preliminary (Radman et al., 2021). However, the issue is important as it determines how participants are classified in terms of language status. For example, some individuals in the “monolingual” group may know two dialects that they do not consider to be separate languages, and so falsely consider themselves to be monolingual. However, proficiency in two dialects has been shown to have similar effects on cognitive performance as does proficiency in two languages (Wang et al., 2017; Antoniou and Spanoudis, 2020).

The present findings also showed that respondents considered that it was important for a language to have a written form to be considered a unique language. This factor interacted with relatedness, such that a language that was related to another one and did not have a writing system was considered less language-like than a language that was unrelated to another language. In an informal sense, the factors of being unlike other languages (unrelated) and having a writing system increased judgments that the system was a unique language. Many languages, including Creole and several Indigenous languages, do not have a written component yet are clearly unique languages (Sandler, 2018), a finding that may have led to some of the anecdotal episodes reported earlier.

The geographic specificity of a system reduced its perception as a unique language if it did not also have a written form. At the same time, geographically specific languages with a written form were still considered to be less language-like than were languages with written forms that were spoken broadly. The presence of a writing system always increases the perception of a system as being a language, but the effect is mitigated by the relation to other languages and the breadth or specificity of the region in which the language is spoken.

### Who Is Bilingual?

Eight features were evaluated for their role in determining whether an individual should be considered bilingual, and all the features had significant impacts on judgments. Generally, individuals were considered to be more bilingual when they learned a second language more recently than distally, when they took immersion rather than core second-language education, when they were more proficient than less proficient in a second language, when they engaged in extensive rather than minimal usage of the second language, when they were actively receptive rather than passively receptive to a second language, when they continued to use their second language after immigration to a foreign-language country, and when there was a writing system in both languages. None of these results are surprising; they reveal that judgments about whether someone is bilingual are based on multidimensional factors that are all continuous in nature.

Participants classified individuals as more bilingual when both systems had a written component than when only one system had a written component. This finding is in line with the result of the language judgments in which the presence of a writing system increased decisions about the system being a language. Therefore, for both questions, the presence of a writing system and competence with the written forms were important, but research on bilingualism rarely reports this information. Similarly, proficiency was also found to be relevant to judgments of bilingualism. Although many studies report second-language proficiency (e.g., Kaushanskaya and Marian, 2009; Oh et al., 2019), there is rarely any mention of proficiency in reading and writing. These factors likely contribute to whether participants are classified as monolingual or bilingual and in turn to cognitive and brain outcomes.

The type of language education also influenced the judgment of how bilingual an individual was considered to be, with higher judgments of bilingualism for more immersive forms of language instruction. Again, this finding may seem intuitive, yet most studies do not report on the educational background of the participants in the sample. Moreover, by including the current age of the hypothetical individual, the present results demonstrated that longer time intervals since that education took place led to lower judgments of bilingualism. This effect was more pronounced in core education than in immersion education conditions.

The various usage patterns experienced by bilinguals have also been shown to contribute to cognitive and brain outcomes (Green and Abutalebi, 2013; Yang et al., 2018; Struys et al., 2019; Bhandari et al., 2020; Wu et al., 2020), although differences in these patterns are rarely discussed in the research. However, the importance of these differences was confirmed in the present results. For example, an individual who has a weekly phone conversation with extended family in their second language was perceived as more bilingual than an individual who speaks a second language with grandma while cooking dinner. The latter scenario may require less active engagement in the second language because cooking requires both working and speaking/listening, whereas a phone call requires more attention to speaking and listening. A more striking nuance of second-language usage relates to immigration and whether individuals continue to use their first language consistently in the new country. In this study, hypothetical individuals who were now 80 years of age and did not consistently use their first language upon arrival in the new country were rated as less bilingual than individuals who continued to use their first language after arrival, despite both groups having 55 years of consistent use in their first language in their home country and 25 years in the new country. In fact, those who did not consistently use their first language upon arrival to the new country at age 55 were only rated a 6.1/10 for how bilingual they were perceived to be, and ~38% of individuals classified these scenarios as 5/10 or less. An individual with 55 years of experience in another language should surely not be classified as monolingual, yet these data suggest that many individuals would classify them as such. This finding again adds noise to the signal when comparing groups of “monolinguals” and “bilinguals.”

Research on the cognitive and brain consequences of bilingualism remains controversial, with studies showing both positive effects of bilingualism and no difference between groups. There are several reasons for null findings (discussion in Bialystok, 2020), but this study suggests that definitions used to determine group membership are potentially a fundamental source of the controversy. As we have seen, there is little consensus about what constitutes a language or what criteria determine whether an individual is bilingual. Both concepts turn out to be complex and multidimensional. Moreover, the present results demonstrated that participants' self-identification as monolingual or bilingual had questionable reliability when evaluated in terms of more objective indicators of bilingual experience. Since many studies rely exclusively on simple self-classification by participants, it may not be surprising that results differ.

The primary implication of the present findings is that between-groups comparisons require clear and objective definitions for the composition of the groups for any interpretations to be made. Variations on the dimensions investigated here can obfuscate true differences between groups by challenging the validity of the group designations. Calling a system a language does not necessarily make it so, and calling an individual bilingual may or may not reflect relevant linguistic experience. But without attention to these definitions, no conclusions can be made about the role of language experience in producing modifications in cognitive or brain systems. Bilingualism is not a categorical variable, and research investigating its multifaceted and complex role in modifying cognitive systems must be clear about the definition. Finally, a detailed description of the bilingual competence of the participants in the sample is an essential first step.

## Data Availability Statement

The raw data supporting the conclusions of this article will be made available by the authors, without undue reservation.

## Ethics Statement

The studies involving human participants were reviewed and approved by York University's Office of Research Ethics. The patients/participants provided their written informed consent to participate in this study.

## Author Contributions

DW, EB, and JG conceptualized and designed the study. DW performed the statistical analyses. DW and JG wrote the first draft of the manuscript, which was then edited by EB. All authors contributed to the manuscript revision, read, and approved the submitted version.

## Funding

The research reported in this article was funded by grant A2559 from the Natural Sciences and Engineering Research Council of Canada to EB. The open-access publication fees for this article were covered by the Iowa State University Library.

## Conflict of Interest

The authors declare that the research was conducted in the absence of any commercial or financial relationships that could be construed as a potential conflict of interest.

## Publisher's Note

All claims expressed in this article are solely those of the authors and do not necessarily represent those of their affiliated organizations, or those of the publisher, the editors and the reviewers. Any product that may be evaluated in this article, or claim that may be made by its manufacturer, is not guaranteed or endorsed by the publisher.
