# Supplementary material for: What Is a Language? Who Is Bilingual? Perceptions Underlying Self-Assessment in Studies of Bilingualism
Source: Front Psychol. 2022 May 12;13:863991. doi: 10.3389/fpsyg.2022.863991 (PMC9134110; doi:10.3389/fpsyg.2022.863991)
Supplement: Supplementary file 1 [file Data_Sheet_1.docx]

**Appendix A**

**Fictional Language Scenarios**

1. (Related, not written)
   You are walking in the park and hear someone speaking Raltan. Raltan is based on French however it does not have a written component. Individuals who speak Raltan can also understand and speak French. Is Raltan a language?
2. (Unrelated, not written)

You walk into a store and hear someone speaking Ragadu. Ragadu is actively spoken but it does not have a written form. Ragadu is not based on any other language – it is completely unique. Is Ragadu a language?

1. (Related, written)
   You are shopping in the grocery store and hear someone speaking Dostinese. Dostinese is similar to English but is written using a different writing system. Individuals who speak Dostinese can also understand and speak English because of the similarities. Is Dostinese a language?
2. (Unrelated, written)
   You are riding on the bus and hear someone speaking Yardish. Yardish is completely unique, not based on any other language but it is written using the familiar Roman alphabet. Is Yardish a language?
3. (Related, geographic, written)

You are sitting in a restaurant and hear someone speaking Andrenia. Andrenia is related to, but distinct from Italian. Andrenia is only spoken in a small geographical area and is written in the same way as Italian. Is Andrenia a language?

1. (Related, geographic, not written)

You are travelling and hear someone speaking Samafu. Samafu is related to, but distinct from Mandarin and is only spoken in a small geographical area. Samafu does not have a written component. Is Samafu a language?

**Appendix B**

**Fictional Bilingualism Scenarios**

1. (Education, core)
   Imagine an individual grew up speaking Jantsi in the home and in the community but from ages 6 to 14 received daily, one-hour lessons in Gronk at school. This individual is now 21 years old. To what extent is that individual bilingual?
2. (Education, immersion)

Imagine an individual grew up speaking Keartian in the home and in the community. Keartian is not like other languages but has a formal written component based on the Roman alphabet. From ages 6 to 17, this individual received a full education in Plinch at school. This individual still uses Keartian to communicate with family and community members. This individual is now 23 years old. To what extent is that individual a bilingual?

1. (Education, extended immersion)

Imagine an individual grew up speaking Hanish in the home and in the community, but from ages 6 to 17 they received a full education in Frunian. Additionally, when this individual was 18, they moved to a country for undergraduate studies where Frunian is the dominant language and all the courses were taught in Frunian. However, this individual still speaks Hanish when they interact with family members. They are now 21 years old. To what extent is this individual a bilingual?

1. (Education, core, age)
   Imagine an individual who grew up speaking Pruchil in the home and in the community but from ages 6 to 14 received daily, one-hour lessons in Brakien at school. This individual is now 48 years old. To what extent is that individual bilingual?
2. (Education, immersion, age)
   Imagine an individual who grew up speaking Botu in the home and in the community. Botu is not like other languages and does not have a formal written component. From ages 6 to 17, this individual received a full education in Mootrilin at school. This individual still uses Botu to communicate with family and community members. This individual is now 37 years old. To what extent is that individual bilingual?
3. (Education, extended immersion, age)
   Imagine an individual who grew up speaking Janish in the home and in the community, but from ages 6 to 17 they received a full education in Pranian. Additionally, this individual moved to a country for undergraduate studies where Pranian is the dominant language, and all the courses were taught in Pranian. However, this individual still speaks Janish when they interact with family members. They are now 51 years old. To what extent is this individual a bilingual?
4. (Middle community use)

Imagine an individual grew up speaking Krental in the home and at school but grew up in an area where Pontal is used in the community. This individual uses Pontal to communicate with members of their community but does not use it otherwise. To what extent is this individual a bilingual?

1. (Most community use)

Imagine an individual grew up in a home with first- or second-generation immigrant parents who mostly speak Ranska at home but also speak some Droot. This individual used Droot at school and a mix of Ranska and Droot in the community. The parents of this individual wanted their children to feel connected to their community, so the individual was sent to Ranska school every Saturday. To what extent is this individual a bilingual?

1. (Passive Receptive language)

Imagine an individual grew up in an environment where both Litsar and Woniva are spoken in the home. However, Litsar is only spoken between this individual’s parents and Woniva is used by the individual to communicate with their parents, siblings and in their community. This individual hears their parents communicating in Litsar but does not use Litsar to communicate. To what extent is this individual a bilingual?

1. (Active Receptive language)

Imagine an individual grew up in a home with parents who mostly speak Mininese at home but also speak some Blonish. This individual’s parents speak Mininese to the individual and the individual responds to the parents in Blonish. To what extent is this individual a bilingual?

1. (Minimal community use)

Imagine an individual grew up in a community surrounded by mostly second and third generation immigrants. In the community, individuals speak Noglish but still use some components of Retruin which was used in their country of origin. To what extent is this individual a bilingual?

1. (Extended family use - cooking with grandma)

Imagine an individual grew up speaking Ceatin in the home, at school, and in the community. However, once a week this individual’s grandmother came over to teach them how to cook and communicated in Ilantin. To what extent is this individual a bilingual?

1. (Extended family use - phone call)
   Imagine an individual who grew up speaking Ganishian in the home, at school, and in the community. However, once a week this individual talks on the phone to their extended family in Devutish. To what extent is this individual bilingual?
2. (Unwritten)

Imagine an individual grew up speaking Mewaki. Mewaki is related to Endranian but does not have a written form. Individuals who speak Mewaki can also speak Endranian since the two languages are similar. To what extent is that individual a bilingual?

1. (Late immigration, discontinued use)
   Imagine an individual immigrated to Canada from a country where Shelinese is the dominant language when they were 55 years old. This individual married someone who speaks a different language. This individual is now 80 years old and has not used Shelinese consistently since leaving their country of origin. To what extent is this individual bilingual?
2. (Late immigration, continued use until recently)
   Imagine an individual immigrated to Canada at 55 years old from a country where Trunish is the dominant language. This individual immigrated with their partner and the two communicated in Trunish for the last 40 years. The individual is now 85 years old. To what extent is this individual bilingual?
3. (Written)
   Imagine an individual grew up speaking Tenilan. Tenilan is related to Oftolish and both are written using the Roman alphabet. Individuals who speak Tenilan can also speak Oftolish because of the similarities. To what extent is that individual a bilingual?
4. (Moderate Proficiency)
   Imagine an individual grew up speaking Pilanch in the home and in the community but from ages 6 to 17 received a full education in Weronish at school, meaning all of their courses were taught in Weronish. This person struggles to hold a full conversation in Weronish without defaulting to using some words in Pilanch. To what extent is that individual a bilingual?
5. (Low Proficiency)
   Imagine an individual grew up speaking Elinese in the home and in the community but from ages 6 to 17 received daily one-hour lessons in Lipnish at school. This person can now only recall a few words in Lipnish. To what extent is this individual a bilingual?
6. (High Proficiency)
   Imagine an individual grew up speaking Chontsi in the home and in the community but from ages 6 to 17 received a full education in Vorozich at school, meaning all of their courses were taught in Vorozich. This person can hold a conversation in Vorozich. To what extent is this individual a bilingual?
